# Very Early-Onset IBD-Associated IL-18opathy Treated with an Anti-IL-18 Antibody

**DOI:** 10.3390/jcm13206058

**Published:** 2024-10-11

**Authors:** Anthea Guha, Rodrigo Diaz-Pino, Andrew Fagbemi, Stephen M. Hughes, Robert F. Wynn, Gloria Lopez-Castejon, Peter D. Arkwright

**Affiliations:** 1 Lydia Becker Institute of Immunology and Inflammation, Manchester M13 9PL, UK; anthea.guha@mft.nhs.uk (A.G.); rodrigo.diaz-pino@manchester.ac.uk (R.D.-P.); gloria.lopez-castejon@manchester.ac.uk (G.L.-C.); 2 Royal Manchester Children’s Hospital, Manchester M13 0JH, UK; andrew.fagbemi@mft.nhs.uk (A.F.); robert.wynn@mft.nhs.uk (R.F.W.)

**Keywords:** VEOIBD, IL-18, IL-18opathy, anti-IL-18

## Abstract

**Background/Objectives**: The aetiology of inflammatory bowel disease (IBD), particularly if occurring early in childhood, is a diverse and patient-focused treatment that is required when standard therapy is ineffective. **Materials and Methods:** A clinical case report is presented of a child with very early-onset IBD (VEOIBD) and evidence of high serum IL-18 responding to anti-IL-18 immunotherapy. Detailed cytokine profiling was performed by ELISA and multiplex assay flow cytometry. **Results:** A four-year-old girl with recalcitrant VEOIBD from six weeks old due to an IL-18opathy, characterised by high blood IL-18 concentration, responded to therapy with a novel neutralising anti-IL-18 antibody (GSK1070806). After two years of hospitalisation, the child’s systemic inflammation and extensive upper and lower gastrointestinal mucosal ulceration remitted with this cytokine inhibitor, allowing the discontinuation of total parenteral nutrition and the resumption of normal oral intake and daily activities. After 18 months on regular GSK1070806, the patient remains in disease remission. **Conclusions:** VEOIBD can be associated with evidence of an underlying IL18opathy and responds to anti-IL-18 antibody therapy. IL-18 should be measured in patients with IBD unresponsive to conventional treatments, and, if elevated, anti-IL-18 antibody therapy should be considered as a potential therapy.

## 1. Introduction

The underlying aetiology of very early-onset inflammatory bowel disease (VEOIBD) is diverse, with over 80 monogenic conditions known to cause a disordered epithelial barrier, impaired mucosal defence, or loss of immune tolerance [1,2]. Standard therapy with 5-aminosalicylates, steroids, immunomodulators, or anti-TNF biologics are often ineffective, necessitating other therapies including Haemopoietic Stem Cell Therapy (HSCT) and non-TNF biologics [3].

IL-18 is an inflammatory cytokine/alarmin produced by bone marrow-derived monocytic cells as well as inflamed mucosal surfaces. Pro-IL-18, like IL-1, is activated by inflammasomes. IL-18 signals via the IL-18Rα/IL-18Rβ heterodimer, expressed mainly on activated T cells, NK cells, and NK-like ILCs, triggering the release of other proinflammatory cytokines, cell proliferation, and cytotoxicity. Its role in human IBD and other inflammatory diseases is still poorly understood [4]. IL-18 gene variants and elevated concentrations of the cytokine have been associated with IBD [5,6]. In children, higher levels of IL-18 and IL-18-Binding Protein (IL-18BP) have been found in Crohn’s disease but not in ulcerative colitis or unclassified IBD [7]. Murine models can provide useful information regarding the aetiology and potential therapeutics for human IBD. An anti-IL-18 monoclonal antibody has been shown to alleviate intestinal inflammation in a murine experimental colitis model [8]. A child with IBD and macrophage activation syndrome (MAS) caused by an NLR family CARD domain-containing protein 4 (*NLRC4)* gain-of-function (GOF) gene variant was reported to respond to recombinant human IL-18BP [9]. Here, we present evidence showing that VEOIBD caused by disordered IL-18 function (IL-18opathy) can be successfully treated with a novel neutralising anti-IL-18 antibody (GSK1070806).

## 2. Materials and Methods

### 2.1. Study Medication

GSK1070806 is a humanised high-affinity IgG1 monoclonal antibody directed against IL-18. It was well tolerated in adults as a single 0.0008–10 mg/kg dose [10], in a phase 2 clinical trial in adults with type 2 diabetes mellitus [11], and in a separate phase 1B clinical trial in adults with moderate-to-severe atopic dermatitis (NCT04975438). It is currently being investigated in a phase 2 study in adults with moderate-to-severe atopic dermatitis (NCT05999799).

There are no previous efficacy or safety data regarding GSK1070806 in children. After GlaxoSmithKline (GSK) reviewed a clinical synopsis presented by the attending physicians of the child’s life-threatening and debilitating disease with no available satisfactory alternative treatment and evidence of excessive IL-18 activity in the patient’s blood, they agreed to supply the drug for compassionate use in our patient. The use of GSK70806 was also formally approved by the Medical Director and the Individual Patient Request Committee of the Hospital Trust. The drug was commenced at a dose of 0.25 mg/kg intravenously every fortnight and then incrementally increased at 3–4 monthly intervals with careful monitoring up to 5 mg/kg fortnightly.

### 2.2. Ethics, Consent, and Drug Approval

The parents provided written consent to use the drug and publish the case within an ethics committee-approved research study (IRAS No. 259418). Consent was also obtained from adult control participants and the parents/legal guardians of participating children as part of a research protocol approved by the NHS Research Ethics Committee.

### 2.3. Measurement of Serum Cytokines

The patient’s blood was tested for inflammatory cytokines. Five healthy adult controls and five children with sepsis-related fevers caused by no underlying primary immune disorder under the care of the Paediatric Haematology and Oncology service at Royal Manchester Children’s Hospital were recruited as controls.

Serum was obtained by centrifuging the blood sample at 1500× *g* for 10 min in a refrigerated centrifuge. The resulting supernatant was collected and transferred to a new tube.

Cytokines were measured using the LEGENDplex human inflammation panel-1 which allows simultaneous quantification of IL-1β, IFN-α2, IFN-γ, TNF-α, MCP-1 (CCL2), IL-6, IL-8 (CXCL8), IL-10, IL-12p70, IL-17A, IL-18, IL-23, and IL-33 (BioLegend, San Diego, CA, USA). Cytokines were quantified using FACSverse (BD Bioscience, Wokingham, UK) and LEGENDplex data analysis software version 8.0. IL-18 concentrations in serum were measured using human total IL-18 DuoSet ELISA (R&D Systems, Minneapolis, MN, USA). ELISAs were performed following the manufacturer’s instructions.

Statistical differences between groups were measured by ANOVA. A two-tailed *p*-value of <0.05 was considered significant.

## 3. Results

### 3.1. Case Presentation

**Figure 1:**
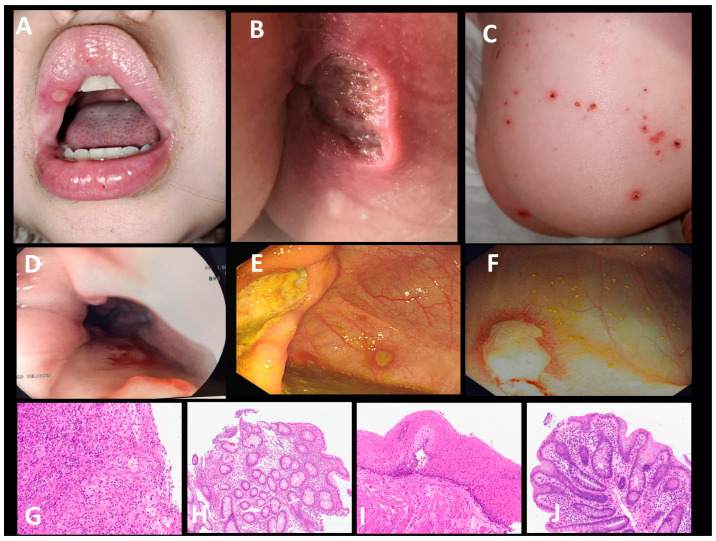
Clinical illustrations of patient’s severe inflammatory disease pre-IL-18 therapy and gut histology pre- and post-IL-18 therapy. (**A**) Perioral ulceration, (**B**) deep perianal ulceration, (**C**) inflammatory skin disease, (**D**) extensive oesophageal ulceration with complete loss of mucosa, (**E**) slough and underlying ulceration of ileocaecal valve, (**F**) colitis, (**G**) oesophageal ulcer denuded of normal squamous epithelium pre-GSK1070806, (**H**) colonic inflammation/ulceration pre-GSK1070806, (**I**) oesophagus with squamous epithelium restored post-GSK1070806, (**J**) colonic mucosa restored post-GSK1070806.

A 4-year-old girl presented at 6 weeks old with recurrent fevers occurring every few days, a scanty papular rash on her face and torso, oral and deep perianal ulceration, severe gastrointestinal mucosal ulceration (Figure 1A–F), and raised C-reactive protein (CRP) (119 mg/L) and faecal calprotectin (600–800 mg/g faeces). Upper and lower endoscopy and biopsies repeatedly showed the extensive mucosal ulceration of the oesophagus, ileocaecal valve, colon, and perianal area (Figure 1G,H). The small bowel (duodenum and ileum) showed only minor inflammation in the lamina propria. A histological analysis of oesophageal samples (ulcer) and colonic mucosa showed a mixed neutrophil, macrophage, and lymphocyte infiltration indicative of inflammation but no granuloma or vasculitis. The oesophageal ulceration was severe enough to involve the underlying muscularis (Figure 1D,G). The child vomited with food, was unable to swallow, and could not be given sufficient nutrition orally. Initially, gastro–jejunal feeds were tolerated but, with time, she was unable to tolerate any enteral feeds and required total parenteral nutrition.

There was no consanguinity or significant family history. The Genomics England R15 primary immunodeficiency and monogenic inflammatory bowel disease gene panel of 429 genes did not reveal any pathogenic variants, including genes known to be associated with high IL-18 levels and inflammation such as NLRC4 or XIAP [12]. Further targeted gene screening did not show any pathogenic intronic, exon, upstream, or downstream flanking region variants in *IL18*, *IL18BP*, *IL18RB*, *IL18R1*, *IL-37*, or *NLRC4*. No abnormalities in *IL18* copy number were detected.

The patient only partly responded to high-dose corticosteroids (1–2 mg/kg/day), and weaning the dose led to disease flares. Treatment with cyclosporine, sirolimus, colchicine, etanercept, infliximab, and anakinra was tried sequentially for 4–6 months each, without any steroid-sparing effect. Because of her recalcitrant disease and lack of improvement with multiple immunosuppressants and biologics, her increasing potential for corticosteroid toxicity and that over half of the patients with VEOIBD are amenable to allogeneic HSCT [13]; at 20 months old, she underwent a 10/10 matched unrelated donor HSCT, using fludarabine (160 mg/m^2^ over 4 days), thiotepa (10 mg/kg over 24 h), treosulfan (42 g/m^2^ over 3 days), and alemtuzumab (1.0 mg/kg over 5 days) as conditioning. She initially had full donor engraftment and disease remission, but at six months post-transplantation, her myeloid donor chimerism progressively fell to <20% and her fevers and her original IBD relapsed.

### 3.2. Cytokine Profiling

In view of the disease relapse, returning to high CRP, we decided to perform a more detailed analysis of the patient’s blood inflammatory cytokine levels using the LEGENDplex human inflammation panel-1 (BioLegend, San Diego, CA, USA), FACSverse (BD Bioscience, Wokingham, UK), and LEGENDplex data analysis software version 8.0. The patient’s results were compared with five healthy adult controls, as well as five age-matched children with sepsis-associated fevers but no underlying primary immunodeficiency (febrile controls: Table 1).

**Figure 2:**
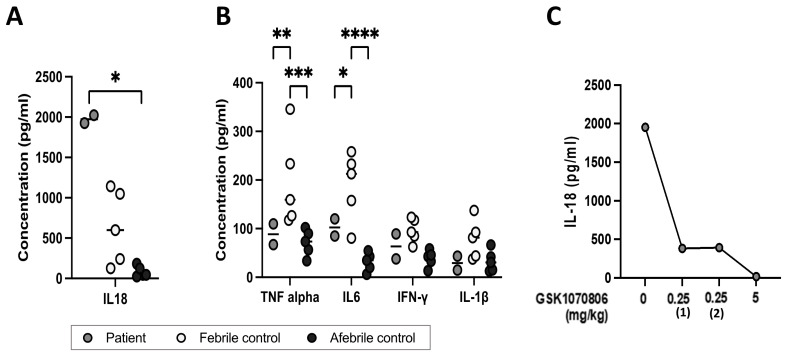
Serum cytokine concentrations in the patient compared to controls. (**A**) Serum total IL-18, (**B**) serum TNF-α, IL-6, IFN-γ, and IL-1β concentrations in patient prior to treatment (two samples taken at different times), febrile children (n = 5), and healthy adult controls (n = 5); (**C**) serum total IL-18 levels in patient prior and after treatment for different days and doses. Kruskal–Wallis and Mann–Whitney U test. * *p*-value < 0.05; ** *p*-value < 0.01; *** *p*-value < 0.005; **** *p*-value < 0.001; if not indicated, data not significant.

The patient had significantly higher serum total IL-18 concentrations than healthy adult controls (HCs) and febrile children (*p* < 0.0002) (Figure 2A). Serum TNF-α, IL-6, IFN-γ, and IL-1β concentrations were not elevated compared to the healthy adult or fever paediatric controls. Febrile controls showed high levels of TNF-α and IL-6, indicating a different inflammatory profile to the patient described here (Figure 2B). IL-12p70, IL-17A, IL-23, IFN-α2, IL-10, and IL-33 were also assayed but not raised.

### 3.3. Impact of Anti IL-18 Therapy

**Figure 3:**
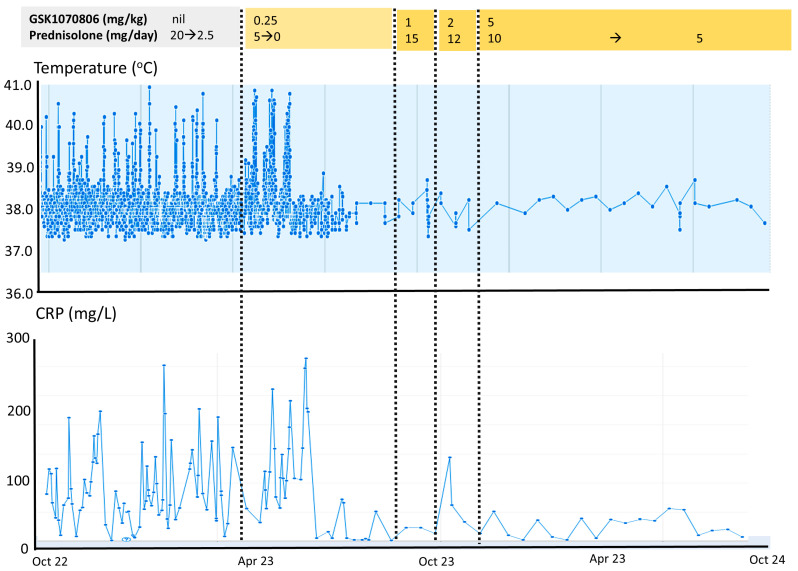
Improvement in fevers and CRP during 18 months of treatment with GSK1070806 neutralising anti-IL-18 antibody. Top legend shows doses of GSK1070806 and prednisolone. Upper panel shows temperature, and lower panel serum CRP concentrations up to and during 18 months of GSK1070806 administration.

In view of the isolated elevated serum IL-18 and failure of other treatments, targeted therapy with an anti-IL-18 biologic was administered. GSK1070806 is a humanised high-affinity IgG1 monoclonal antibody directed against IL-18. As this biologic has not been previously administered in children, it was commenced fortnightly at a dose of 0.25 mg/kg intravenously, and it was incrementally increased at 3–4 monthly intervals to 5 mg/kg. At a dose of 1 to 5 mg/kg fortnightly, the patient’s fevers, gastrointestinal symptoms, faecal calprotectin (decreased from 600 to 800 mg/g faeces pre-treatment to a nadir of 134 mg/g faeces on 5 mg/kg GSK1070806), and CRP settled, allowing for the discontinuation of total parental nutrition, the resumption of a normal diet, and discharge from hospital (Figure 3 shows the resolution of fevers and improvement in serum CRP). A histochemical analysis of upper and lower endoscopy biopsies on 5 mg/kg of GSK1070806 showed the restoration of the squamous epithelium in the post-oesophagus as well as the restoration of the colonic mucosa, demonstrating that the inflammatory changes—particularly marked in the oesophagus, ileocecum, and colon—had largely been resolved (Figure 1I,J). Oral steroids were reduced to 0.3 mg/kg/day. Total serum IL-18 was undetectable on 5 mg/kg of GSK1070806 (Figure 2C). There were no adverse effects relating to the drug at any stage of treatment.

## 4. Discussion

This study demonstrates the clear sustained response to 18 months of administration of anti-IL-18 antibody therapy in a child with VEOIBD and high total serum IL-18 concentrations. The clinical improvement correlated with the neutralisation of serum total IL-18 by the anti-IL-18 monoclonal antibody in vivo. The clinical phenotype and clinical response of our patient mirror the previously reported case of a child with an *NLRC4* GOF gene variant who responded to anti-IL-18BP [9]. In our patient, despite detailed genetic analysis, no known pathogenic variant could be found in this gene. IL-37 inhibits IL-18 signalling by binding to IL-18R1/IL-1R8 heterodimers. IL37 deficiency has also been associated with VEOIBD, but again, no pathogenic *IL37* gene variants were found in this patient [14]. It may be that a somatic gene mutation underlies this patient’s disease and only genomic analyses of specific cell types, e.g., cell-sorted monocytes or tissues, e.g., intestinal mucosa, will elucidate the underlying pathogenic gene variant.

High IL-18 levels have been found in systemic Juvenile Idiopathic Arthritis (sJIA) and Adult-Onset Still’s Disease (AOSD), particularly when associated with macrophage activation syndrome (MAS) (summarised in a review by Shimizu et al. [15]). Likewise, we saw high IL-18; however, we did not observe increases in other proinflammatory cytokines, including IL-12, as IL-18 works in conjunction with IL-12, via IL-18R, to induce IFNγ [16]. However, the increase in IL-18, but not IL-12, would explain the lack of IFN-γ observed in our patient’s serum, as well as the absence of MAS.

Recombinant IL-18BP (tadekinig alfa) has previously been found to be safe in a small phase II open-label trial (NCT02398435) of ten adults with AOSD and for compassionate use in two adults suffering from AOSD [17,18]. Larger clinical trials are required to draw any definite conclusions regarding efficacy. It has also been used to treat a boy with XIAP deficiency and haemophagocytic lymphohistiocytosis (HLH), who presented with high serum levels of IL-18 [19]. As an X-linked condition, XIAP deficiency is primarily a disease found in males, but a few case reports have been published with female patients [20]. In our female patient, no pathogenic *XIAP* variants were detected, ruling out this condition.

The mechanisms of the cellular release of IL-18 are very similar to that of IL-1β [21]. Unlike proIL-1β, proIL-18 is stored in most cells but particularly bone marrow-derived monocytic cells and barrier epithelia (skin and respiratory and gastrointestinal tracts) [22]. Traditionally, cleavage is mediated by caspase-1 upon sensing a wide range of danger and pathogenic stimuli. The effect of broad-spectrum antibiotics on the gut flora may explain the transient improvement in fevers and CRP despite the persistently negative blood cultures and bacterial 16S RNA in our patient prior to anti-IL-18 therapy. Its release can also be triggered by the alternative inflammasome and mediated by caspase-4 upon sensing intracellular lipopolysaccharide [23]. Deficiency of this gene would be expected to reduce, rather than increase, IL-18 activity.

Although other non-steroidal immune modulators and biologics were ineffective, we observed a transient clinical response after HSCT until donor myeloid engraftment was lost. The loss of donor engraftment and the similarity in histology pre- and post-transplant to the original disease, rather than graft-versus-host disease, strongly suggest disease recurrence rather than a complication of HSCT. As we could not completely rule out the possibility that gut, as well as monocyte-derived IL-18, was contributing to this patient’s disease, rather than pursuing a second HSCT, we opted for anti-IL-18 therapy, which would be easy to discontinue if adverse effects occurred. This therapy led to an excellent clinical benefit in terms of the remission of systemic and gut inflammation and improved the quality of life for the patient and her family. Corticosteroids continue to be weaned successfully with the higher GSK1070806 dose. We envisage that the anti-IL-18 therapy will need to be continued long-term to maintain remission. If the GSK1070806 were to become less effective, for instance, because of neutralising antibodies, treatment options include (i) increasing the drug dose to 10 mg/kg, (ii) switching to an alternative anti-IL-18 biologic, or (iii) reconsidering a second HSCT.

## 5. Conclusions

This study describes VEOIBD with evidence of high total serum IL-18 concentrations and clinical response to anti-IL-18 antibody therapy. It highlights the importance of measuring IL-18 in patients with autoinflammatory diseases unresponsive to conventional treatments. Clinical trials of GSK1070806 or the IL18BP antagonist (tadekinig alfa) in IBD and monogenic IL-18-driven autoinflammatory diseases such as sJIA and AOSD should provide further evidence for targeting IL-18 in IL-18opathies [24,25].

## Figures and Tables

**Table 1:** Clinical features of febrile ward paediatric patients.

Gender	Age	Diagnosis	Cause of Fever
Female	2 years	B-cell acute lymphoblastic leukaemia	Viral URTI
Female	6 years	Ewings sarcoma	Viral URTI
Male	6 years	Medulloblastoma	Viral URTI
Male	4 years	Rhabdomyosarcoma	Line infection
Male	20 months	Hepatoblastoma	Line infection

URTI: upper respiratory tract infection.

## Data Availability

Requests for data can be emailed to peter.arkwright@manchester.ac.uk.
